# *Parabacteroides distasonis* ameliorates ETEC-induced diarrhea in weaned piglets by regulating intestinal barrier function and intestinal inflammation

**DOI:** 10.1186/s40813-026-00525-1

**Published:** 2026-06-18

**Authors:** Zichen Wu, Longlin Zhang, Hongkun Li, Zihao Zhang, Bie Tan, Jing Wang

**Affiliations:** 1 Hunan Provincial Key Laboratory for the Products Quality Regulation of Livestock and Poultry, College of Animal Science and Technology, Hunan Agricultural University, Changsha, 410128 China; 2 Yuelushan Laboratory, Changsha, Hunan 410128 China; 3 National Center of Technology Innovation for Dairy, Hohhot, Inner Mongolia 010000 China; 4 The Cotton and Sericultural Research Institute of Hunan Province, Changde, China

**Keywords:** *P. distasonis*, Intestinal barrier, Intestinal inflammation, ETEC, Weaned piglets

## Abstract

*Enterotoxigenic Escherichia coli* (ETEC) is the main pathogen causing bacterial diarrhea in piglets. Ningxiang piglets (native Chinese breed) has the characteristics of stress resistance and low diarrhea rate, which is closely related to the intestinal microbial structure shaped by its high fiber feed. Our study found that *Parabacteroides distasonis* (*P. distasonis*), a representative intestinal bacterial species in Ningxiang piglets, had a positive correlation with fecal secretory immunoglobulin A (sIgA). *P. distasonis* strain was isolated from Ningxiang piglets and verified the regulation on intestinal injury induced by ETEC in piglets. Results showed *P. distasonis* could alleviate the weight loss of piglets caused by ETEC infection. *P. distasonis* significantly decreased the diarrhea rate and inhibited the colonization of ETEC in intestinal mucosa, while decreased the ileal mucosal IL-1β, IL-6 and TNF-α levels after ETEC infection (*P* < 0.05). *P. distasonis* decreased the serum DAO activity, D-lactate and Endotoxin and upregulating the expression of small intestine mucosal sIgA. *P. distasonis* enhanced the expression of tight junction proteins, including claudin-1, claudin-3, occludin, and zonula occludens-1 (ZO-1), at both the transcriptional and translational levels (*P* < 0.05). It also upregulates the expression of intestinal epithelial proliferation markers including *PCNA*, *β-catenin*, *CHGA*, *Lgr5* and *LYZ* (*P* < 0.05). Furthermore, *P. distasonis* affected the microbial structure and increased the relative abundances of beneficial genera, as well as increased the SCFAs contents in ileum of ETEC-challenged piglets. Taken together, *P. distasonis* could maintain the integrity of intestinal epithelial barrier, alleviate intestinal inflammation and repair intestinal barrier damage caused by ETEC, which provides theoretical basis for nutritional intervention of diarrhea in piglets.

**Supplementary Information:**

The online version contains supplementary material available at https://doi.org/10.1186/s40813-026-00525-1 .

## Introduction

Post-weaning diarrhea is an important factor affecting the survival rate of piglets, which increases high morbidity and mortality in swine industry [1]. ETEC infection is the main factor causing diarrhea in piglets after weaning, which mainly causes human and animal diarrhea through intestinal mucosal adhesion and colonization and toxin production [2]. Antibiotics have traditionally been used in animal husbandry to prevent and treat pathogen infections, but the widespread use of antibiotics has led to the emergence of drug-resistant pathogens and drug residues in animal products, which seriously endanger human health [3]. Therefore, in the context of the “total ban on antibiotics”, it is particularly important to explore antibiotic alternatives for preventing and treating early post-weaning piglet diarrhea, as well as improving animal intestinal health.

Gut microbiota are critical for host health and disease, including maintaining intestinal barrier, regulating nutrient absorption, immune response and other physiological processes [4, 5]. Early weaning in piglets induces a loss of gut microbial diversity and intestinal dysbiosis, which significantly increases the risk of intestinal diseases and poses a severe threat to piglet health [6]. Notably, the weaning period may serve as a key window for shaping the gut microbiota [7]. Local pig breeds exhibit high adaptability to specific environmental conditions, and their long-term consumption of high-fiber diets promotes the formation of a unique gut microbiota structure. Ningxiang pig, a local pig breed native to Hunan Province, China, is characterized by low diarrhea incidence in piglets and high disease resistance [8]. Studies have suggested that probiotics can shape a beneficial bacterium-dominant gut microbiota and reduce the potential for pathogen colonization in the gut, thereby providing a viable strategy for preventing and treating intestinal diseases [9]. Thus, we hypothesized that the characteristic differential gut microbes of Ningxiang piglets may act as potential probiotics to improve piglet diarrhea.

In this study, we found that *Parabacteroides distasonis* (*P.distasonis*), a differential fecal microbe of lactating Ningxiang piglets, exhibited a positive correlation with fecal secretory immunoglobulin A (sIgA) levels. *P. distasonis* strain was isolated from Ningxiang piglets, and we intend to explore its regulatory effects on intestinal barrier function and injury repair via piglet models.

## Methods

### Animal studies and experimental design

This study included two animal experiments. In the two breeds of piglets, fecal samples were collected from 8 Ningxiang piglets and 11 Yorkshire piglets at day 14, while 7 Ningxiang piglets and 13 Yorkshire piglets at day 21.The study was conducted at the Yongxing Pig Farm of Hunan Chuweixiang Agriculture & Animal Husbandry Co., Ltd. (Changsha, China) and at Sifanghong farm (ZhangJiakou, China). Through 16 S rRNA sequencing analysis, the main differential bacterial species were identified.

The second animal experiment was a piglet intervention study conducted under a 2 × 2 factorial experiment. A total of 24 21-day-old crossbred weaned barrows (Duroc × Yorkshire × Landrace) were selected and randomly allocated to four treatment groups including the CON group, ETEC group, *P. distasonis* group, and *P. distasonis*+ETEC group. The basal diet was provided by Hunan Lifeng Biotechnology Co., Ltd., with its composition and nutritional levels detailed in Supplementary Table S1. Prior to the formal experiment, all piglets underwent a 3-day adaptation. From days 1 to 14 of the experiment, piglets in the CON and ETEC groups were gavage with 10 mL of sterile normal saline daily, while those in the *P. distasonis* and *P. distasonis*+ETEC groups received a daily gavage of 10 mL *P. distasonis* resuspension (1 × 10⁹ CFU/mL).On day 15, the piglets in the ETEC and *P. distasonis*+ETEC groups were orally challenged with 10 mL of ETEC K88 solution (1 × 10⁹ CFU/mL). Meanwhile, the CON group continued with its original treatment and the *P. distasonis* group maintained its pre-treatment protocol. From days 15–17, the piglets in the *P. distasonis* and *P. distasonis*+ETEC groups continued to receive a daily gavage of 10 mL *P. distasonis* resuspension. In contrast, the CON and ETEC groups were gavaged with 10 mL of sterile normal saline daily using the same method. All piglets had free access to feed and water. Throughout the entire experiment, the piglets were housed in individual pens, with the room temperature maintained at 25 °C and the humidity in the pig house ranging from 50% to 80%.

### Sample collection

On day 18, piglets were euthanized and dissected. Serum samples were collected in anticoagulant-free centrifuge tubes. Subsequently, ileal tissue, ileal mucosa, ileal contents, and terminal colon contents were collected from the piglets, snap-frozen in liquid nitrogen and stored at -80 °C. Parts of the ileal segments were fixed in 4% paraformaldehyde at room temperature for 24 h for subsequent observation and measurement of intestinal morphology. All husbandry practices and experimental procedures were approved by the Institutional Animal Care and Use Committee of Hunan Agricultural University (Changsha, Hunan, China).

### Isolation of *Parabacteroides distasonis* strains and culture conditions

The *Parabacteroides distasonis* HNAU0205 (*P. distasonis*) was originally isolated from the feces of healthy Ningxiang piglets. In brief, fecal samples were serially diluted with sterile phosphate-buffered saline, then isolated and purified on Bacteroides Bile Esculin solid medium at 37 °C for 48 h. Single colonies were picked and identified by 16 S rRNA gene sequence. The isolated strain has been deposited in the China Center for Type Culture Collection (CCTCC, Accession No: M20221529, Wuhan, Hubei, China). *P. distasonis* was cultivated in GAM medium (No. HB8518-1, Qingdao), supplemented with hemin (5 mg/mL) and 1% vitamin K₁ solution, and incubated at 37 ℃ for 24 h under anaerobic conditions. Bacterial counting was performed using Columbia agar medium (No. HB0124-1, Qingdao) supplemented with 5% sterile sheep blood.

### ETEC K88 culture conditions

ETEC K88 (O149:K91, K88ac; LT, STb, EAST) was kindly provided by Prof. Wenkai Ren from South China Agricultural University [10]. ETEC was cultured in LB medium at 37 ℃ for 12 h to reach saturation (≥ 1.0 × 10^9^CFU).

### Growth performance and diarrhea incidence analysis

In this experiment, piglets were weighed on days 0, 7, 14, and 17 of the experiment to analyze their growth performance. Concurrently, the fecal score of each piglet was recorded daily (scoring criteria: 0, normal feces; 1, mild diarrhea; 2, moderate diarrhea; 3, severe diarrhea), The diarrhea rate was determined as the percentage of the total number of diarrheal piglet cases during the experiment relative to the total number of observation cases (days×piglets) [11].

### Biochemical analysis

According to the manufacturer’s instructions, the concentrations of sIgA and β-defensin2 (DEFβ2) in piglet feces were determined using commercial enzyme-linked immunosorbent assay (ELISA) kits from Cusabio Biotech. The concentration of D-lactate (D-LA) and the activity of diamine oxidase (DAO) in serum, as well as the sIgA concentration in ileal mucosa, were measured using corresponding enzyme immunoassay kits according to the manufacturer’s protocol (Ruixinbio, Quanzhou, Fujian, China). The content of endotoxin (ET) in serum was determined using a commercial lipopolysaccharide limulus assay kit (Xiamen Bioendo Technology Co., Ltd., Xiamen, China). For the quantification of cytokine levels in ileal mucosa, a commercial porcine cytokine multiplex immunoassay kit (RayBiotech, Norcross, GA, USA) was used, and cytokine concentrations were calculated using QAP-CYT-1-SW software (RayBiotech, Peachtree Corners, GA, USA).

### RNA isolation and quantitative real-time PCR

The total RNA was extracted using the RNeasy Mini kit according to the instructions (Qiagen, Germany). RNA was inverted into cDNA using PrimeScript™ RT Reagent (TaKaRa, Japan) kit. qRT-PCR was performed using TB Green ™ Premix Ex Taq ™ II (TaKaRa, Japan) combined with A7500 Fast Real-Time PCR system (Applied Biosystems, Foster City, CA, USA) real-time fluorescence quantitative PCR instrument. Relative gene expression level was calculated by comparative CT method (^ΔΔCt^ method) and normalized to *β-actin* housekeeping genes, all primer sequences are listed in Supplementary Table S2.

### DNA isolation and quantitative real-time PCR

As the previously reported [12], total DNA was extracted from ileal mucosa and feces samples using a Stool DNA isolation Kit according the manufacturer’s instructions (SENO Biotech Co., Ltd., Zhangjiakou, China) and DNA concentration was measured using NanoDropND-1000 (Thermo Fisher Scientific, Waltham, MA). The primers targeting the 16 S rRNA gene (Tsingke Biotechnology Co., Ltd., Bejing, China) of *P.distasonis* (5′-GGACACGTCCCGCACTTTAT-3′ and 5′-TTCTGAGAGGAAGGTCCCCC-3′) and ETEC (5′-CATGCCGCGTGTATGAAGAA-3′ and 5′-CGGGTAACGTCAATGAGCAAA-3′). The standard curves were prepared using nine standard concentrations ranging from 100 to 108 gene copies/µL. Amplification was performed with LightCycler (Roche) to obtain melt curve data. Data were analyzed using 2nd Derivative Max (Roche). The copy number was corrected for DNA concentration and the number of 16 S rRNA genes encoded in the *P.distasonis* and ETEC genome.

### Western blot analysis

Ileum tissue was homogenized in 1 mL of RIPA lysis buffer (containing protease and phosphatase inhibitors) on ice. The homogenate was then incubated at 4 °C overnight and was centrifuged at 13,000×g/4°C for 15 min. Use BCA (Beyotime, Shanghai, China) protein concentration assay kit to detect the protein concentration of each sample. Add 5× protein loading buffer and heat at 95 ℃ for 10 min. The same amount of protein was loaded onto the sodium dodecyl sulfate–polyacrylamide gel electrophoresis (SDS-PAGE), and then printed on the polyvinylidene fluoride membrane (PVDF). After sealing the membrane with Quick Block™ sealing solution (Beyotime, Shanghai) for 1 h, it was incubated the primary antibodies for β-actin (1:1000, HUABIO, R1207), ZO-1 (1:500, AFFinity, AF5145), Claudin-1 (1:1000, Absin, abs146616), Occludin (1:1000, Abcam, ab216327), ChgA (1:2000, Abcam, ab283265), Lgr5 (1:2000, OriGene Technologies, TA503316), β-catenin (1:2000, Abcam, ab305261), and PCNA (1:500, HUABIO, SY12-07) overnight at 4℃. Following a 25 min TBST wash, membranes were incubated with the appropriate HRP-conjugated secondary antibody (1:5000, ZSGB-BIO, ZB-2306) for 2 h at room temperature. Protein bands were then detected using an ECL kit (Beyotime, Shanghai), imaged with a chemiluminescence imaging analyzer (Bio-Rad, chemiDoc), and quantified using ImageJ-1.52a software. The relative expression of each target protein was calculated by normalizing its band intensity to that of β-actin.

### Histological analysis and Mucus staining

Paraffin sections were dewaxed in xylene, rehydrated through a graded ethanol series, and subjected to HE staining. After hematoxylin staining, sections were differentiated, rinsed with distilled water, and counterstained with eosin. Under a Zeiss microscope at 40× magnification, five representative fields were randomly selected from each section. Villus height and crypt depth at these positions were measured using ImageJ-1.52a software. Villus height was defined as the vertical distance from the villus tip to the villus - crypt junction, and crypt depth was defined as the depth from the crypt opening to the base. All measurements are presented in micrometers (µm).

AB-PAS staining was performed with Alisin Blue (Solarbio, Beijing, China) in 8 min, Schiff in 15 min and Hematoxylin in 1 min, and then the nuclei were differentiated with acid differentiation solution, and then the nuclei were retreated with Scott Blue for 3 min. Finally, the nuclei were sealed with neutral resin and observed under microscope.

### Immunofluorescence and quantification

Tissue samples were embedded in paraffin and cut into 5 μm thick sections. The sections were permeabilized with 0.1% Triton X-100 and blocked with 5% BSA at room temperature. After three washes with PBS, the sections were incubated overnight with PCNA primary antibody at 4 °C. FITC-conjugated goat anti-rabbit IgG antibody (Beijing Zhongshan Jinqiao Biotechnology Co., Ltd., Beijing, China) was then added, followed by incubation at room temperature for 1 h. The samples were subsequently counterstained with DAPI at room temperature for 10 min. After being washed with PBS three times, the sections were mounted with an anti-fading reagent (Boster Biological Technology Co. Ltd., Wuhan, China). Observation was performed using a laser scanning confocal microscope (ZEISS, LSM 880), and ImageJ-1.52a software was used for image analysis.

### 16S rRNA analysis

Total microbial DNA was extracted from ileal mucosa samples using a Stool DNA Isolation Kit according to the manufacturer’s instructions (SENO Biotech Co., Ltd., Zhangjiakou, China). The V4 hypervariable region of the bacterial 16S rRNA gene was amplified by PCR using the forward primer 515 F (5’-GTGYCAGCMGCCGCGGTAA-3’) and the reverse primer 806R (5’-GGACTACHVGGGTWTCTAAT-3’). PCR products were verified by 2% agarose gel electrophoresis, purified with AMPure XT beads (Beckman Coulter Genomics, Danvers, MA, USA), and quantified using a Qubit fluorometer (Invitrogen, USA). After individual quantification, amplicons were mixed in equal amounts. Paired-end 2 × 250 bp sequencing was performed on the Illumina MiSeq platform with the NovaSeq 6000 SP Reagent Kit at Hangzhou LC-Bio Technologies Co., Ltd. (Hangzhou, China). The average effective sequencing depth was approximately 40,000 clean reads per sample, and all samples had more than 35,000 valid sequences, ensuring sufficient species coverage for subsequent analysis.

### SCFAs analysis

The concentration of short-chain fatty acids in ileal contents was determined by gas chromatography. Briefly, as previously described, approximately 1 g of ileal chyme was homogenized with 1.5 mL of deionized water. The homogenate was then centrifuged at 15,000 × g for 10 min at 4 °C, and 1 mL of the supernatant was collected. The supernatant was mixed with 25% metaphosphoric acid solution at a ratio of 1:5, followed by acidification on ice for 30 min. Finally, the treated sample was injected into a GC 8890 Series gas chromatograph (Agilent Technologies, Santa Clara, CA, USA) for analysis.

### Statistical analysis

The raw data are classified and processed by Excel software (Microsoft, Washington USA). Statistical analysis was performed using ImageJ, SPSS 26.0, and GraphPad Prism 9.5. The data were analyzed using two - factor analysis of variance to examine the main effects of the two treatments (1. Whether to gavage *P. distasonis*; 2. Whether to challenge with ETEC) and their interaction. When a significant interaction between the two treatments was found, a simple main effect analysis was then performed, with post-hoc comparisons corrected for multiple comparisons using the Bonferroni method. The chi - square test was used to evaluate the differences in diarrhea among piglets in each group. Data were represented as Mean ± SEM, the difference is considered significant when the *P*-value is lower than 0.05 (*P* < 0.05). Bioinformatic analysis was performed using the OmicStudio tools at https://www.omicstudio.cn/tool, and online software TUTU analysis platform (https://www.cloudtutu.com/).

## Results

### Key bacterium *Parabacteroides* showed a significant positive correlation with fecal sIgA

**Fig. 1:**
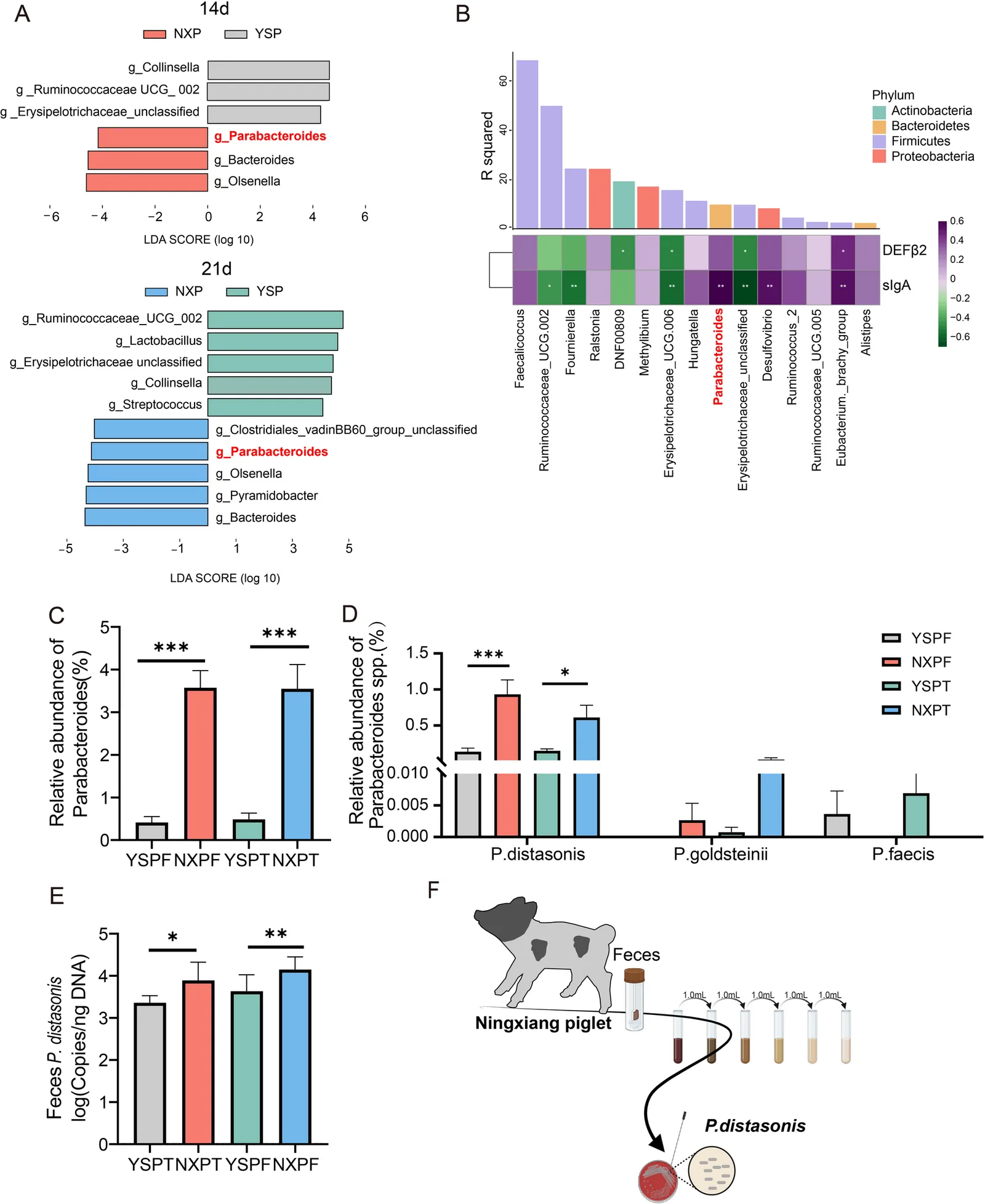
*P. distasonis* was enriched in the intestines of suckling Ningxiang piglets. (**A**) Linear discriminant analysis effect size (LEfSe) analysis showing differentially abundant gut microbes between suckling Ningxiang and Yorkshire piglets, with a linear discriminant analysis (LDA) score > 4 (*P* < 0.01). (**B**) Correlation analysis between intestinal immune indexes (sIgA and DEFβ2) and fecal microbiota composition. (**C**) Relative abundance of the genus *Parabacteroides* in the feces of piglets from different groups. (**D**) Relative abundance of representative strains within the genus *Parabacteroides*, including *P. distasonis*, *P. gordonii*, and *P. faecis*. (**E**) Relative abundance of fecal *P. distasonis* verified by qPCR. (**F**) Isolation of *P. distasonis* strain from the feces of Ningxiang piglets. Data are presented as the Mean ± SME. Statistical significance: “*”indicated 0.01 ≤ *P* ≤ 0.05, “**” indicated 0.001 ≤ *P* ≤ 0.01, and “***” indicated *P* ≤ 0.001. Abbreviations: NXPF, Ningxiang piglets at 14 days of age; YSPF, Yorkshire piglets at 14 days of age; NXPT, Ningxiang piglets at 21 days of age; YSPT, Yorkshire piglets at 21 days of age

To screen the sources of gut microbes associated with anti-infection, we selected healthy Ningxiang piglets and Yorkshire piglets at day 14 and day 21 collected fecal samples to analyze and screen the core biomarker. The LEfSe analysis showed that the bacterial genera, such as *Parabacteroides*, *Bacteroides*, and *Olsenella*, were dominated in Ningxiang piglets at d 14 and 21 of age (Fig. 1A). The fecal sIgA and DEFβ2 levels in Ningxiang piglets were higher than those in Yorkshire piglets (*P *< 0.05) (Fig. S1A-B). We further analyzed the correlation between gut microbiota and fecal anti-infectious indicators (sIgA and DEFβ2) at d 14 and 21 of age. Figures 1B and S1C showed that the abundance of specific gut microbe were positively correlated with the fecal sIgA and DEFβ2 levels of piglets, such as *Ruminococcaceae UCG_002* (14 d and 21 d), *Erysipelotrichaceae_unclassified* (14 d), and *Parabacteroides* (14 d). Noteworthily, among the dominated bacterial genera of Ningxiang piglets, the *Parabacteroides* was significantly positive with fecal sIgA, whereas others were significantly negative with fecal sIgA. We determined the abundances of *Parabacteroides* and its main species *P. distasonis*, *P. goldsteinii* and *P. faecis* in the feces of piglets. The results showed that the relative abundance of *Parabacteroides* in Ningxiang piglets, especially *P. distasonis*, were higher than those in Yorkshire piglets at 14d and 21d (*P *< 0.05) (Fig. 1C-D). Furthermore, we quantified the absolute abundance of *P. distasonis* and found a higher presence of *P. distasonis* in Ningxiang piglets (*P*<0.05) (Fig. 1E). Consequently, *P. distasonis* was identified as a key bacterium and isolated from Ningxiang piglets' feces for further probing into its function on the gut health(Fig. 1F).

### Gavage of *P. distasonis* ameliorated diarrhea and protected against ETEC-challenged intestinal barrier injury

**Fig. 2:**
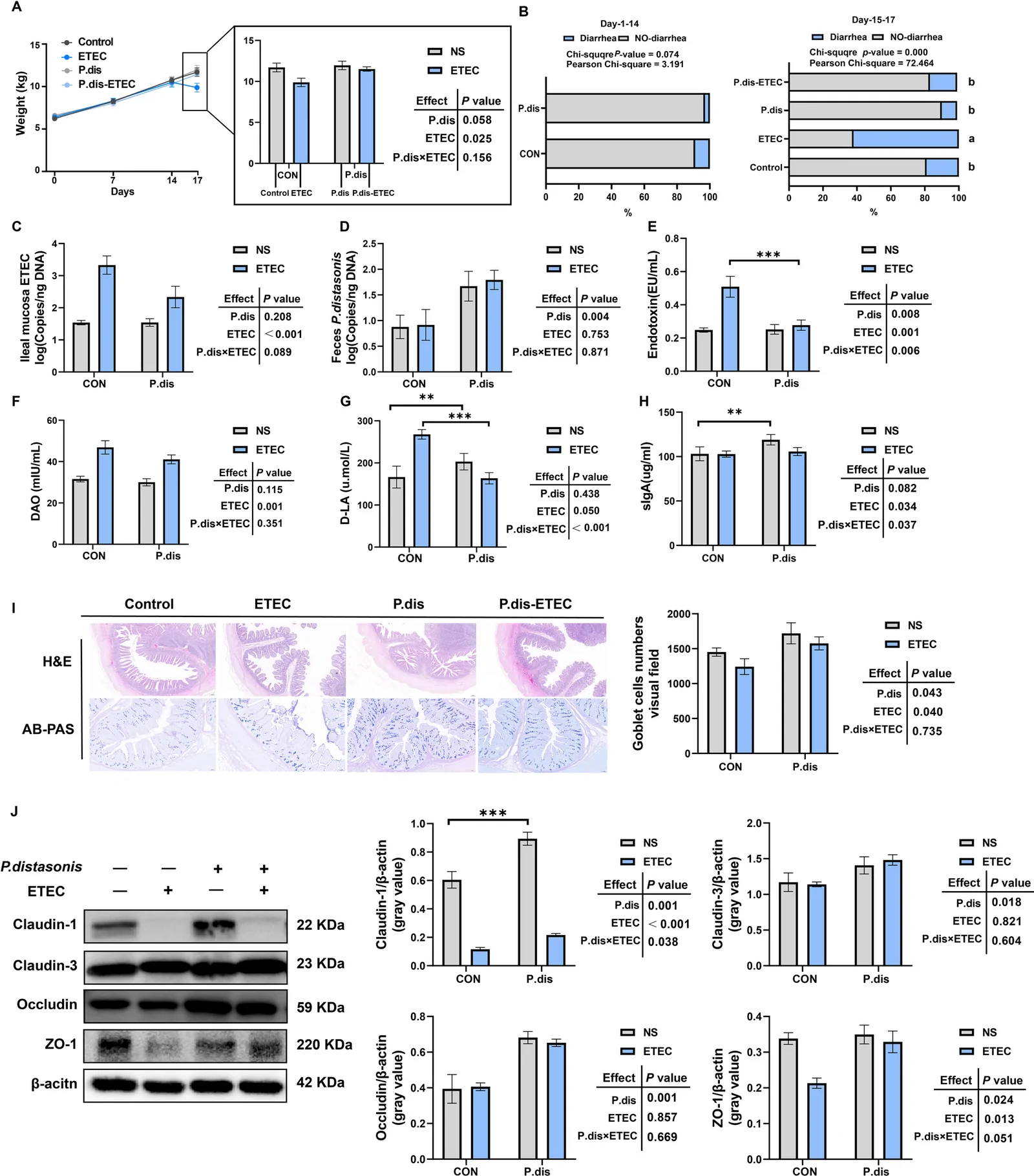
Effects of *P. distasonis* gavage on growth performance and intestinal tight junction protein expression in weaned piglets after ETEC challenge. (**A**) Body weight changes of piglets (P. dis = *P. distasonis*). (**B**) Diarrhea rate of piglets before and after ETEC challenge. (**C**) Relative abundance of ETEC in the ileal mucosa, determined by qPCR. (**D**) Relative abundance of *P. distasonis* in feces, determined by qPCR. (**E**) Serum ET concentration. (**F**) Serum DAO activity. (**G**) Serum D-LA concentration. (**H**) sIgA levels in the ileal mucosa. (**I**) Ileal intestinal morphology visualized by H&E staining and AB/PAS staining (scale bar = 200 μm). (**J**) Protein expression levels of tight junction proteins (Claudin-1, Claudin-3, Occludin, and ZO-1) in the ileum of piglets (*n* = 3). Notes: “+” indicates supplementation with *P. distasonis* or ETEC; “-” indicates no supplementation with *P. distasonis* or no ETEC. Data are presented as the Mean ± SME. “*”indicated 0.01 ≤ *P* ≤ 0.05, “**” indicated 0.001 ≤ *P* ≤ 0.01, and “***” indicated *P* ≤ 0.001

During the pre-challenge period, *P. distasonis* treatment had no significant effect on piglet body weight (Fig. 2A). On the 3rd day post-challenge, *P. distasonis* treatment tended to increase piglet body weight (*P* = 0.058), whereas ETEC challenge significantly reduced piglet body weight (*P* < 0.01). In the pre-challenge period, *P. distasonis* treatment tended to decrease the piglet diarrhea rate. *P.distasonis* effectively decreased the diarrhea rate of piglet after ETEC challenge (*P* < 0.01) (Fig. 2B). By quantifying the presence of ETEC in the ileum mucosal and contents of different groups, we found that the ETEC-challenged group yielded a large population of ETEC (*P* < 0.001) in ileum mucosa compared with the Control group (Figs. 2C, S2A). However, when pre-treated with *P.distasonis* as preventive measure before the ETEC-challenged, demonstrating a notable reduction of ETEC was observed in ileum mucosal and content of piglets (Figs. 2C, S2A). Moreover, *P. distasonis* was enriched in the feces of piglets in the *P.distasonis*-treated groups (*P* < 0.01) (Fig. 2D).

Previous studies have shown that ETEC challenge can damage the intestinal barrier, lead to the destruction of tight junction proteins and increase intestinal permeability, thus causing diarrhea. In our study, *P. distasonis* supplementation significantly decreased the serum ET level (*P* < 0.01), while ETEC challenge markedly increased the serum ET (*P* < 0.001) and DAO levels (Fig. 2E-F). There were interactions between *P. distasonis* supplementation and ETEC challenge on serum ET and D-LA levels (Fig. 2E-G). Compared with the Control group, the concentrations of serum ET and D-LA were significantly higher in piglets from the ETEC group (*P* < 0.001). No significant differences in serum ET and D-LA levels were observed between the *P. distasonis*-treated groups and the Control group (Fig. 2E-G). Additionally, supplementation of *P. distasonis* had a trend of increased ileum mucosal sIgA level (*P* = 0.082), while ETEC challenge decreased the sIgA level (*P* < 0.05). There was no significant interaction between supplementation of *P. distasonis* and ETEC challenge on sIgA level (*P* > 0.05) (Fig. 2H).

Morphological analyses revealed that ETEC challenge led to intestinal injury, including ileal villi shrinkage and shedding (*P* < 0.001) (Fig. 2I). The villus height (VH) and ratio VH/CD in ileum of ETEC-challenged piglets were significantly decreased (*P* < 0.001). Supplementation of *P. distasonis* reduced the crypt depth (CD) and increased the ratio VH/CD in ileum (*P* < 0.001) (Figs. 2I, S2B-D). Notably, there was interaction between supplementation of *P. distasonis* and ETEC challenge on the VH, crypt depth (CD) and the ratio of VH/CD. AB-PAS staining results showed that ETEC challenged significantly reduced the number of goblet cells in the ileum of piglets, and *P. distasonis* supplementation markedly increased the number of goblet cells (*P* < 0.001). (Fig. 2I). Analysis of intestinal tight junction components revealed that *P. distasonis* supplementation significantly upregulated the mRNA expression of *Claudin-3* (*P* < 0.01), *Occludin* (*P* < 0.05), and *ZO-1*(*P* < 0.001), whereas ETEC challenge downregulated the expression of *Claudin-1*(*P* < 0.01) and *Occludin*(*P* < 0.05) (Figure S2E-H). This pattern was corroborated at the protein level. The protein abundances of ZO-1 (*P* < 0.05), Occludin (*P* < 0.001), and Claudin-3 (*P* < 0.05) were significantly increased in the *P. distasonis*-treated group compared to the untreated control(Fig. 2J). Conversely, ETEC challenge reduced the protein levels of Claudin-1 and ZO-1 (both *P* < 0.05). A significant interaction between *P. distasonis* and ETEC was specifically observed for Claudin-1 protein expression (*P* < 0.05), indicating that the probiotic modulated the pathogen-induced change in this particular junction protein (Fig. 2J).

### Gavage of *P. distasonis* reduces ETEC-challenged the expression of inflammatory cytokines

**Fig. 3:**
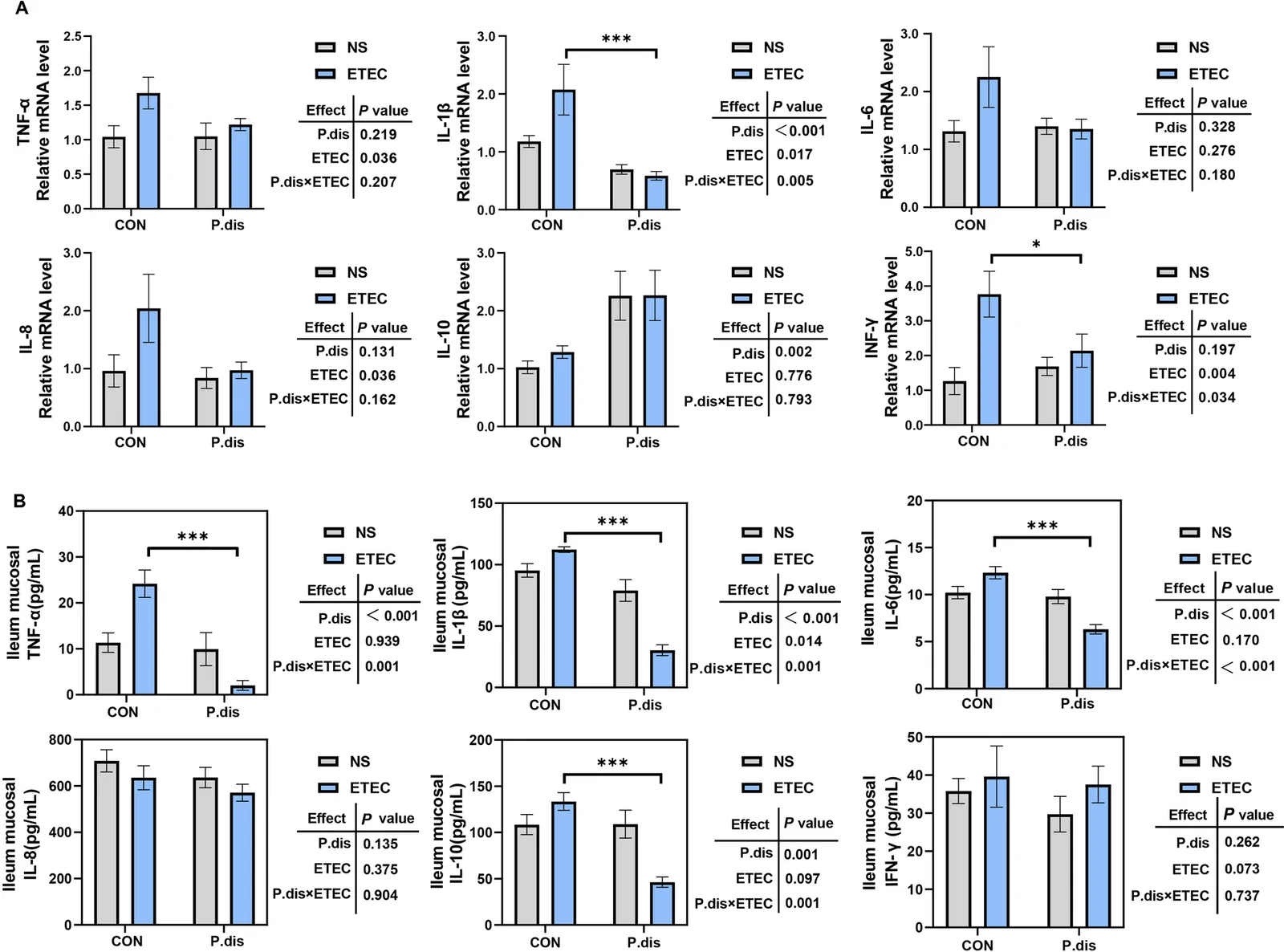
Effects of *P. distasonis* gavage on intestinal cytokine levels in weaned piglets after ETEC challenge. (**A**) Relative mRNA expression levels of inflammatory cytokines (*TNF-α*, *IL-1β*, *IL-6*, *IL-8*) and anti-inflammatory cytokine (*IL-10*) as well as interferon-γ (*IFN-γ*) in the ileum. (**B**) Concentrations of TNF-α, IL-1β, IL-6, IL-8, IL-10, and IFN-γ in the ileal mucosa. Data are presented as the Mean ± SME. “*”indicated 0.01 ≤ *P* ≤ 0.05, “**” indicated 0.001 ≤ *P* ≤ 0.01, and “***” indicated *P* ≤ 0.001

As showed in Fig. 3, ETECchallenge significantly upregulated the mRNA expressions of *TNF-α* (*P* < 0.05), *IL-1β*(*P* < 0.05), *IL-8* (*P* < 0.05) and IFN-γ(*P* < 0.05), while supplementation of *P. distasonis* significantly altered the mRNA expressions of *IL-1β* (*P* < 0.001) (Fig. 3A). There was a significant interaction between supplementation of *P. distasonis* and ETEC challenge on the *IL-1β* and *IFN-γ* (*P* < 0.05). ETEC challenge induced *IL-1β* and *IFN-γ* expression in control piglets, while this induction was markedly attenuated in *P. distasonis*-pretreated animals(Fig. 3A). In contrast, the anti-inflammatory cytokine *IL-10* (*P* = 0.002) was significantly increased in supplementation of *P. distasonis*, indicating that *P. distasonis* supplementation significantly and constitutively upregulated IL-10 expression irrespective of infection status (Fig. 3A). No significant interaction or main effect of ETEC was found for *IL-10*. There was no notable difference in the mRNA expressions of *IL-6* in ileum among four groups (*P* > 0.05) (Fig. 3A). To further evaluate the actual impact of *P. distasonis* on local intestinal inflammation, we measured the protein concentrations of key cytokines in the ileal mucosa (Figure B).There were significant interactions between supplementation of *P. distasonis* and ETEC challenge on intestinal mucosal pro-inflammatory cytokines TNF-α, IL-1β and IL-6 levels in the ileum. Meanwhile, there were no significant interactions IL-8 and IFN-γ levels (*P* > 0.05), suggesting a specific immunomodulatory profile of *P. distasonis* that does not interfere with these pathways(Fig. 3B). There was significant interaction between supplementation of *P. distasonis* and ETEC challenge on the anti-inflammatory cytokine IL-10 levels (*P* < 0.05). Post-hoc analysis revealed that *P. distasonis* significantly reduced IL-10 levels in ETEC-challenged pigs compared with the infected control group (Fig. 3B).

### Gavage of *P. distasonis* promotes ISC proliferation and differentiation in ETEC-challenged piglets

**Fig. 4:**
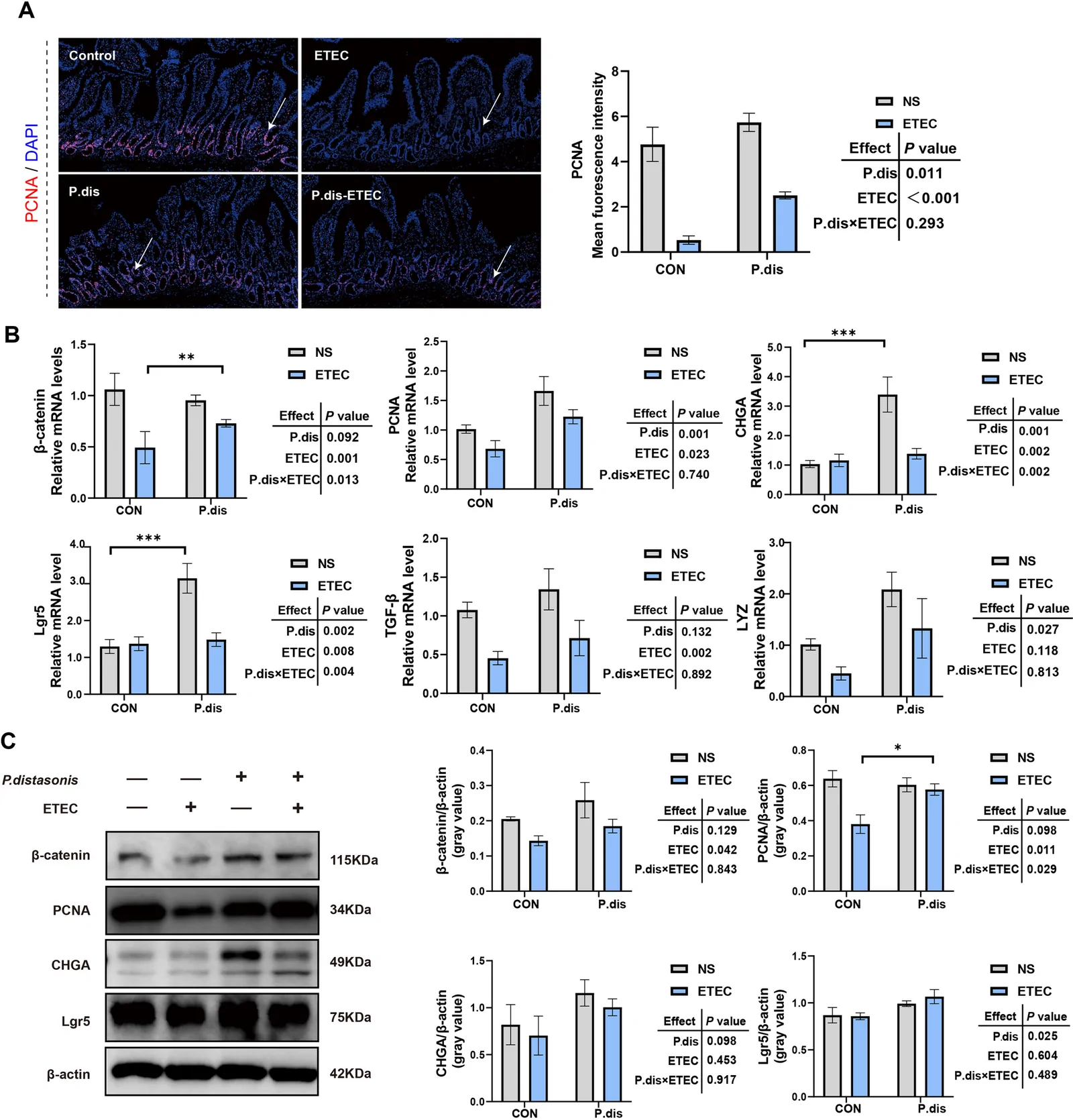
Effects of *P. distasonis* gavage on intestinal development in weaned piglets after ETEC challenge. (**A**) Immunofluorescence staining for PCNA in piglet. (**B**) Relative mRNA expressions of *β-catenin*, *PCNA*, *CHGA*, *Lgr5*, *TGF-β* and *LYZ* in piglet (*n* = 6). (**C**) Protein expressions of β-catenin, PCNA, CHGA and Lgr5 in piglet ileum (*n* = 3). Notes: “+” indicates supplementation with *P. distasonis* or ETEC; “-” indicates no supplementation with *P. distasonis* or no ETEC challenge. Data are Mean ± SME. “*”indicated 0.01 ≤ *P* ≤ 0.05, “**” indicated 0.001 ≤ *P* ≤ 0.01

The immunofluorescence results showed that ETEC challenge significantly reduced the number of PCNA⁺ cells in the ileal crypts, whereas supplementation with *P. distasonis* independently increased PCNA⁺ cell counts (Fig. 4A). There was no significant interaction between supplementation of *P. distasonis* and ETEC challenge, indicated that the probiotic’s effect on epithelial proliferation was independent of infection status. ETEC challenge consistently downregulated the expression of *β-Catenin* (*P* < 0.01), *PCNA *(*P* < 0.05), *CHGA* (*P* < 0.01), *Lgr5*(*P* < 0.01) and *TGF-β* (*P* < 0.01), while supplementation with *P. distasonis* significantly upregulated the expression of *PCNA*, *CHGA* and *Lgr5* (*P* < 0.01).There were significant interactions between supplementation of *P. distasonis* and ETEC challenge on the *β-Catenin*, *CHGA and Lgr5* (*P* < 0.05), indicating that *P.distasonis* altered the host transcriptional response to infection (Fig. 4B). ETEC challenge inhibited *TGF-β* expression, while *P. distasonis* supplementation elevated *LYZ* levels (*P* < 0.05). There were no significant interactions between *TGF-β* and *LYZ* (Fig. 4B). Additionally, ETEC challenge significantly decreased β-catenin and PCNA protein levels (*P* < 0.05) (Fig. 4C). There was a significant interaction between *P. distasonis* supplementation and ETEC challenge on PCNA, the inhibitory effect of ETEC was attenuated in *P. distasonis*-treated piglets. Independently, *P. distasonis* supplementation significantly increased Lgr5 protein expression (*P* < 0.01). No significant changes were detected in CHGA protein expression across groups (Fig. 4C).

### Gavage of *P. distasonis* affected the gut microbial composition in ETEC-challenged piglets

**Fig. 5:**
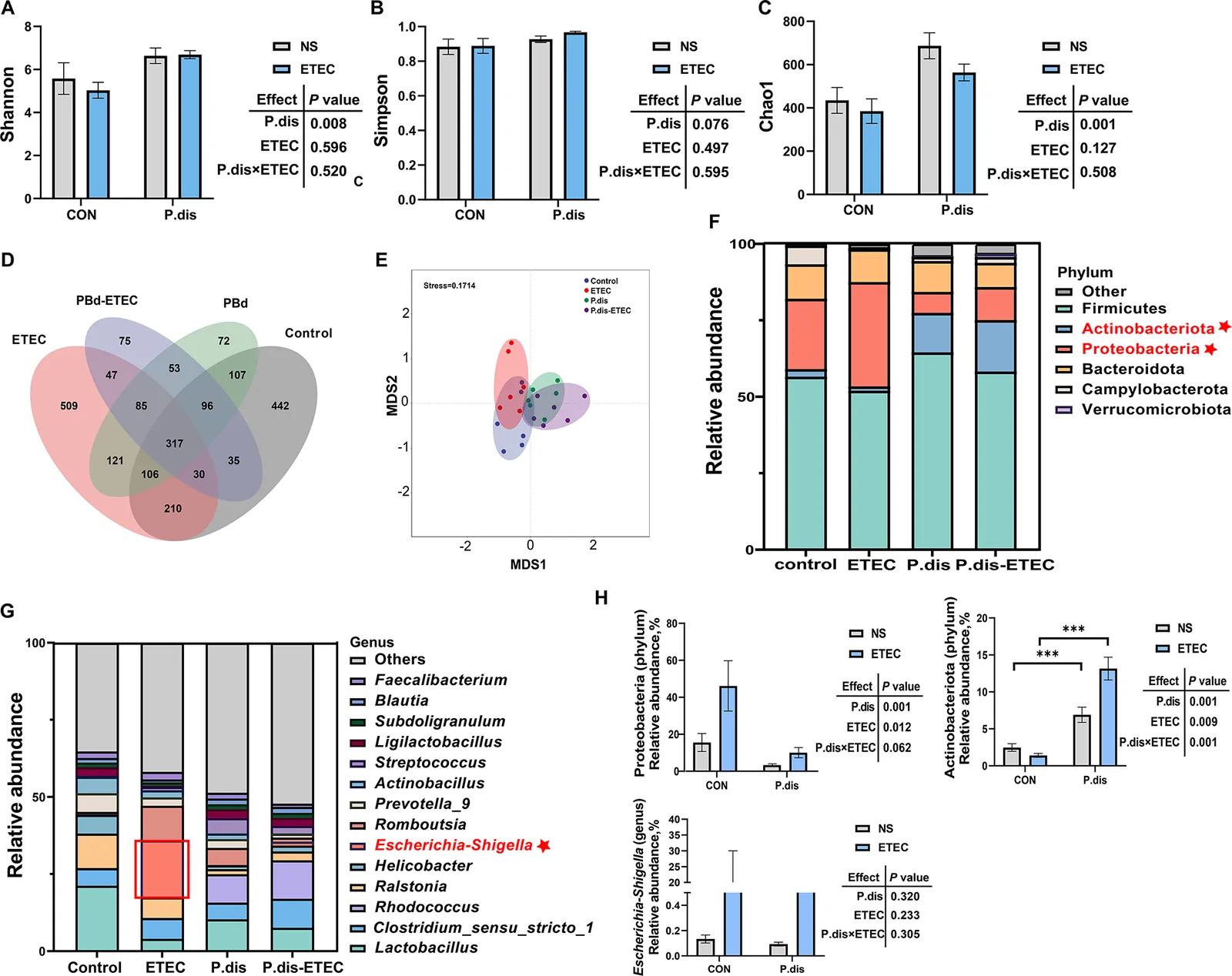
Effects of *P. distasonis* gavage on intestinal microbial diversity in weaned piglets after ETEC challenge. (**A**-**C**) α-diversity indices including Shannon, Simpson and Chao1. (**D**) OTUs of ileal mucosal microbiota. (**E**) Non-Metric Multi-Dimensional Scaling (NMDS) showing ileal mucosal microbiota profiles among four groups. (**F**) Stacked column chart showing the relative abundance of top 6 phyla. (**G**) Stacked column chart showing the relative abundance of top 15 genera. (**H**) The relative abundance of Proteobacteria, Actinobacteriota, and *Escherichia-Shigella. *Data are Mean ± SME. “***” indicated *P* ≤ 0.001

Different gut microbiota interdependence and mutual restraint to form a dynamic balance, which provides a protective barrier for intestinal tract. *P. distasonis* treatment significantly increased the Shannon index (*P* < 0.01) and Chao1 index (*P* < 0.01) compared to the CON group (Fig. 5A, C). In the absence of *P. distasonis*, ETEC infection no significant difference was observed in the Shannon index(Fig. 5B). No significant interaction was found between *P. distasonis* supplementation and ETEC challenge for any of the indices. A total of 2305 core operational taxonomic units (OTUs) were identified (Fig. 5D). As shown in NMDS plot, the P.dis-ETEC group formed a distinct cluster clearly separated from the ETEC group (Fig. 5E). Moreover, the gut microbiota showed obvious segregation among CON, P.dis and P.dis-ETEC groups (Fig. 5E).

Regarding the phylum differences, the Firmicutes and Proteobacteria were dominated phyla in piglets, followed by Bacteroidetes and Actinobacteriaote (Fig. 5F). Nonetheless, the relative abundances of Proteobacteria (*P* < 0.05) were increased by ETEC-challenged piglets(Fig. 5H), while the relative abundances of Actinobacteria was increased markedly after oral administration of *P.distasonis* (*P* < 0.001) (Fig. 5H). At the genus level, the ETEC-challenged increased the relative abundances of *Escherichia-Shigella* (Fig. 5G, H), which was agreement with the trends observed in the absolute concentrations of ETEC (Figs. 2D, H).

### *P. distasonis* raised SCFAs concentration of intestinal content

**Fig. 6:**
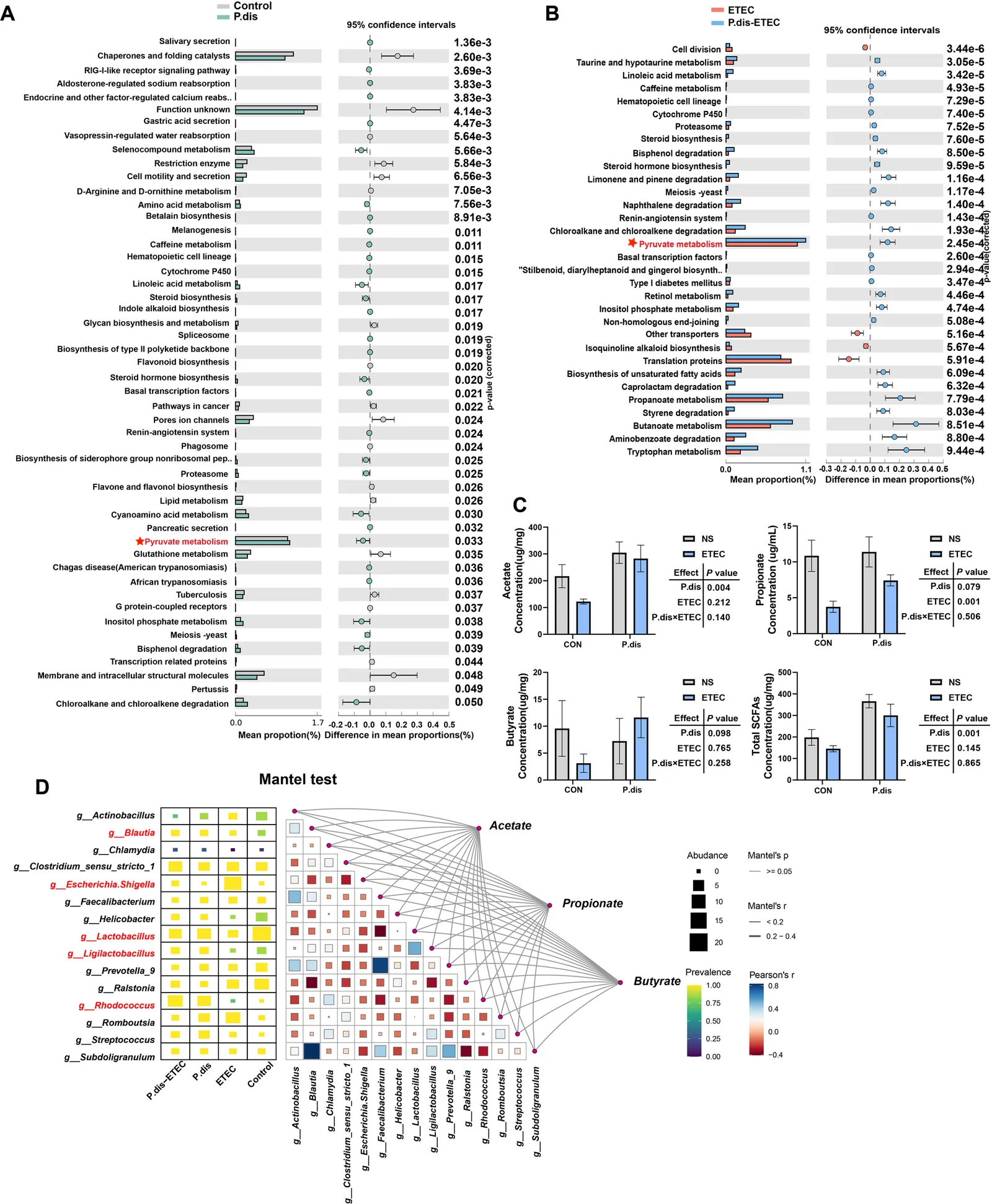
Effects of *P. distasonis* gavage on SCFA concentrations in intestinal contents of weaned piglets after ETEC challenge. (**A**) The differentially represented metabolic pathways at the Kyoto Encyclopedia of Genes and Genomes (KEGG) level 3 through stamp analysis between the CON and P.dis group (*P* < 0.05). (**B**) Differences in metabolic pathways in ETEC and P.dis-ETEC group (*P* < 0.001). (**C**) The concentration of acetate, propionate, butyrate and SCFAs in ileum of piglets. (**D**) The results of association analysis between SCFAs and ileum specific bacterial genera, respectively. Data are presented as the Mean ± SME

We further compared the abundance of KEGG genes related to pathways between the Control vs. P.dis group and ETEC vs. P.dis-ETEC group. Our result indicated a significantly higher abundant of pyruvate metabolism after supplementation of *P.distasonis*, especially compared to the ETEC group (Fig. 6A-B). Consequently, we further measured the SCFAs concentrations. The concentrations of acetate (*P* < 0.01) and total SCFAs (*P* < 0.001) were significantly increased and the concentrations of propionate (*P* = 0.079) and butyrate (*P* = 0.098) showed increasing trends after *P.distasonis* supplementation, while ETEC-induced significantly decreased on the concentrations of propionate (*P* < 0.001) (Fig. 6C). There were no significant interactions between supplementation of *P.distasonis* and ETEC challenge on the acetate, propionate, and butyrate, suggesting that the promoting effect of *P. distasonis* was not specifically altered by ETEC challenge. We next assessed the potential correlation relationship between SCFAs and gut microbiota which was influence by *P.distasonis*. The top 15 genus (higher relative abundance), with higher prevalece, was screened to further analysis, with the *P.distasonis* supplementation, the proportion of *Lactobacillus*,* Ligilactobacillus* and *Blautia* were increased in ETEC-challenged piglets (Fig. 6D). Mantel correlation analysis revealed that there were had no significantly correlation with microbiota and SCFAs (Fig. 6D).

## Discussion

Gut microbiota are involved in shaping host immune system and regulating intestinal immune development [13, 14]. Compared with Yorkshire piglets (foreign lean meat breeds), *P. distasonis* was identified as a representative strain in the intestinal tract of Ningxiang piglets and was significantly positively correlated with fecal sIgA levels. *P. distasonis* is a Gram-negative bacterium belonging to the genus *Parabacteroides*, which commonly colonizes the gastrointestinal mucosa of numerous species and plays important roles in host intestinal tract [15]. Studies showed that *P. distasonis* effect on treating inflammatory bowel disease (IBD) [16] and inflammatory arthritis [17]. *P. distasonis* also regulates host metabolism dysfunction [18] and ameliorate insulin resistance [19]. However, different host sources and strain types of *P.distasonis* would affect its function. In the present study, we isolated *P. distasonis* from Ningxiang piglets, and evaluated its effect on the intestinal barrier function and ETEC infection resistance in piglet model.

It has been reported that *P. distasonis* is a potential next-generation probiotic [15]. Consistent with previous studies, *P. distasonis* could improve the growth performance of animals [20]. Our study shows that ETEC infection leads to a significant decrease in the body weight of piglets, while supplementation with *P. distasonis* could reduce the weight loss in ETEC-challenged piglets. ETEC K88 is a major cause of diarrhea in weaned piglets [21]. Consistent with previous reports [3, 22, 23], we found that the diarrhea rate of piglets in the ETEC group was higher than in the Control group, while significantly lower in *P. distasonis*-treated groups. Meanwhile, administration of *P. distasonis* was found significantly reduced the colonization of ETEC in the ileal mucosa. The above results indicate that supplementation with *P. distasonis* could alleviate ETEC-induced growth retardation, while inhibiting the colonization and proliferation of ETEC and reducing the incidence of diarrhea.

The intestinal barrier is necessary for maintaining the homeostasis of the intestinal environment and resisting pathogen invasion. Diarrhea always accompanied by intestinal barrier structure damage [24]. As expected, our result found that the ETEC infection resulted in the destruction of the intestinal integrity of piglets and led to intestinal injury, while gavage of *P.distasonis* significantly reversed these changes. Besides, administration of *P. distasonis* not only significantly increased the number of intestinal goblet cells in healthy piglets, but also significantly elevated this index in ETEC-infected piglets, which is consistent with previous studies [3, 25]. The reversal of the aforementioned conditions following the administration of *P. distasonis* suggests its potential regulatory role in intestinal immunity. The present study found that ETEC infection reduced the level of sIgA in the intestinal mucosa of piglets, whereas *P. distasonis* significantly increased the intestinal mucosal sIgA level in healthy piglets. sIgA plays an important role in the prevention and clearance of ETEC infections [26]. McKenzie et al. demonstrated that sIgA produced after ETEC infection could inhibit the colonization of ETEC and reduce its ability to cause diarrhea [27]. This result suggests that supplementation with *P. distasonis* may represent an effective strategy for enhancing intestinal mucosal immunity.

Elevated serum DAO and D-lactate levels are usually associated with impaired intestinal barrier function [28]. In this study, *P. distasonis* administration significantly reduced the levels of DAO and D-lactate in serum of ETEC-infected piglets, and alleviated the intestinal injury induced by ETEC. ET is a special component on the cell wall of *Escherichia coli*, which is the main antigen in ETEC-induced diarrhea [10]. Our results showed that supplementation with *P. distasonis* could significantly decrease serum endotoxin contents in ETEC-challenged piglets. Meanwhile, *P. distasonis* significantly increased the expression of tight junction proteins weaned piglets. The decreased expressions of *ZO-1*, *Claudin-1*, and *Occludin* are associated with intestinal barrier dysfunction and increased permeability [29]. The above results indicate that *P. distasonis* could reduce intestinal permeability and block the endotoxin translocation, thereby alleviating ETEC-induced diarrhea, which was consistent with previous research on probiotics [17–19, 30, 31].

Increased intestinal permeability leads to abnormal activation of subepithelial immune cells and excessive production of inflammatory factors [32]. Studies have consistently identified the pro-inflammatory cytokines TNF-α and IL-8 as markers for inflammatory responses induced by E. coli and LPS [21], whereas IL-10 is an anti-inflammatory cytokine that suppresses immune responses [33]. In the present study, ETEC infection upregulated the expression of genes encoding *TNF-α* and *IL-8* in the ileum of piglets, and supplementation with *P. distasonis* reversed this effect. Consistent with previous studies, *P. distasonis* and its membrane components could inhibit the expression of pro-inflammatory factors (IL-1β, IL-8, and TNF-α) [34–36]. Meanwhile, *P. distasonis* supplementation upregulated the expression of the gene encoding *IL-10* in the ileum of weaned piglets, which aligns with the findings of Cuffaro et al., who demonstrated that *P. distasonis* promotes the repair of intestinal damage following ETEC infection by enhancing *IL-10* expression to counteract or inhibit ETEC [31]. Interestingly, our results revealed a discrepancy between IL-10 levels and its gene expression in the ileal mucosa. This discrepancy could be attributed to the multi-step regulation from “genetic signals” to “functional proteins”, mRNA exhibits a rapid response, while protein response is delayed, resulting in a “temporal mismatch in expression peaks” between the gene and its corresponding protein [37]. IFN-γ is a cytokine involved in host defense against viral infections and in regulating Th1 responses [38]. Our data showed that ETEC infection upregulated IFN-γ expression in the ileum in weaned piglets, whereas *P. distasonis* supplementation reduced IFN-γ levels in ETEC-infected piglets. Collectively, these results indicate that *P. distasonis* supplementation can alleviate ETEC-induced intestinal inflammation.

Unquestionably, the integrity of intestinal morphology and function relies on continuous intestinal epithelium regeneration. ISCs are the foundation of intestinal epithelial proliferation and could support intestinal regeneration [39]. Previous studies suggested that Lgr5^+^ stem cells could maintain tissue homoeostasis and induced intestinal regeneration after injury [40]. In the current study, *P. distasonis* repaired the proliferation ability of intestinal stem cells after intestinal injury, which is supported by the increased expression levels of Lgr5 and PCNA in the small intestine in piglets. Besides promoting proliferation, *P.distasonis* also promoted ISCs differentiate into Paneth cells. Paneth cells play an important role in maintaining ISCs proliferation by secreting EGF, TGF and LYZ [39, 41]. Our results showed that ETEC infection downregulates the mRNA expression of *TGF-β*, while *P.distasonis* upregulated the mRNA expression of *LYZ* in weaned piglets. The wnt/β-catenin signaling pathway is essential for maintaining the homeostasis of intestinal epithelial stem cells [42]. In this study, we further found that *P. distasonis* significantly increased the protein expression of β-catenin in piglets to increase the accumulation of β-catenin to support proliferation, which is necessary to repair intestinal injury caused by ETEC infection. CHGA secreted by intestinal endocrine cells has been proven to be involved in regulating the permeability of the intestinal barrier [43]. Our results suggest that *P.distasonis* significantly up-regulates the mRNA and protein expression of CHGA and attenuated the ETEC-induced loss of intestinal absorptive cells, which may contribute to the restoration of normal intestinal barrier function. In conclusion, supplementation with *P*. *distasonis* could upregulate the expression of intestinal epithelial proliferation markers and key repair pathways, and is accompanied by morphological improvement of ETEC-induced epithelial damage. These findings suggest that *P*. *distasonis* may contribute to the restoration of intestinal epithelial homeostasis following ETEC infection.

Pathogen infection causes an imbalance of gut microbiota, which has adverse effects on the physiological functions of the body, leading to inflammatory enteritis and diarrhea. Previous studies have shown that the increase in gut microbiota diversity may enhance the stability of gut microbiota, thereby enhancing the resistance to pathogens [44]. Studies have shown that ETEC infection significantly reduces intestinal diversity and causes gut microbiota imbalance [45]. In our study, *P. distasonis* increased the microbial species in the ileal mucosa of normal piglets, and alleviated the diversity reduction caused by ETEC challenges. Our results also showed that the relative abundance of the Proteobacteria phylum increased after by ETEC challenged, which is consistent with previous studies [3, 46]. *P. distasonis* significantly decreased the presence of coliforms in the ileal mucosa. This result was confirmed by the absolute quantification of ETEC in ileal mucosa, and a consistent result was obtained. It has been reported that diarrhea piglets show a decrease in *Lactobacillus* and an increase in the abundance of *Escherichia coli* [47]. Our results support this conclusion, and *P. distasonis* supplementation could significantly reversed this dysbiosis. Through microbial functional prediction, we found *P. distasonis*/ETEC induced changes in microbial function. It mainly enriched pathways related to pyruvate metabolism, which was a direct metabolic precursor for SCFA production via microbial fermentation. Accordingly, measurement of SCFAs in ileal contents revealed that ETEC challenge significantly reduced propionate, whereas *P. distasonis* supplementation increased acetate. Acetate plays a crucial role in gut homeostasis by modulating immune cell function to resolve inflammation and by enhancing epithelial barrier integrity [48]. Thus, our findings suggest that *P. distasonis* may alleviate post-ETEC intestinal damage via this acetate-mediated dual mechanism, thereby promoting mucosal repair. Interestingly, our results revealed that none of the top 15 bacterial genera correlated with any of the three SCFAs (acetate, propionate, and butyrate). This suggest that SCFA levels may not be directly attributable to a few dominant taxa, but rather represent the integrated metabolic product of the entire microbial community. Furthermore, these levels are significantly modulated by the host’s rapid intestinal absorption and utilization [49]. Together, these observations reflect the complexity of spatial heterogeneity and functional redundancy within the gut ecosystem [50].

## Conclusion

This study indicates that *P. distasonis* could serve as a potential probiotic for preventing diarrhea in early-weaned piglets. *P. distasonis* was capable of alleviating ETEC-challenged diarrhea in weaned piglets, promoting intestinal repair after injury, preserving the integrity of the intestinal epithelium, and reducing inflammatory responses in piglets. Meanwhile, *P. distasonis* could restore the balance of the gut microbiota, enhance the symbiosis between beneficial bacteria, and inhibit the proliferation of pathogenic bacteria. While the probiotic was administered via oral gavage in this controlled study, its efficacy and safety when delivered through feed or drinking water warrant further evaluation for field application. Additionally, the precise molecular targets and signaling pathways through which *P. distasonis* repairs ETEC-induced intestinal injury require further elucidation.

## Supplementary Information

Below is the link to the electronic supplementary material.


Supplementary Material 1


## Data Availability

The 16 S rRNA sequencing data for piglets from two breeds are publicly available under NCBI BioProject ID PRJNA976087.

## Abbreviations

ETEC: *Enterotoxigenic Escherichia coli*
*P.distasonis*: *Parabacteroides distasonis*
CFU: Colony forming unit
sIgA: Secretory immunoglobulin A
DAO: Diamine oxidase
ET: Endotoxin
D-LA: D-lactic acid
NC: Normal Saline
TNF-α: Tumor necrosis factor-α
ZO-1: Zona occludens 1
IL: Interleukin
MUC2: Mucin 2
IFN-γ: Interferon-γ
ISC: Intestinal stem cells
PCNA: Proliferating Cell Nuclear Antigen
TGF-β: Transforming growth factor-β
Lgr5: Leucine-rich repeat- containing G protein-coupled receptor 5
CHGA: Chromogranin A
NMDS: Non-metric Multidimensional Scaling

## References

[1] Campbell JM, Crenshaw JD, Polo J. The biological stress of early weaned piglets. J Anim Sci Biotechnol. 2013;4:19. 10.1186/2049-1891-4-19. 23631414 PMC3651348

[2] Fleckenstein JM, Hardwidge PR, Munson GP, Rasko DA, Sommerfelt H, Steinsland H. Molecular mechanisms of enterotoxigenic Escherichia coli infection. Microbes Infect. 2010;12:89–98. 10.1016/j.micinf.2009.10.002. 19883790 PMC10647112

[3] Li M, Zhao D, Guo J, Pan T, Niu T, Jiang Y, et al. Bacillus halotolerans SW207 alleviates enterotoxigenic Escherichia coli-induced inflammatory responses in weaned piglets by modulating the intestinal epithelial barrier, the TLR4/MyD88/NF-κB pathway, and intestinal microbiota. Microbiol Spectr. 2024;e0398823. 10.1128/spectrum.03988-23. PMC10986599 38451226

[4] Zhou B, Yuan Y, Zhang S, Guo C, Li X, Li G, et al. Intestinal Flora and Disease Mutually Shape the Regional Immune System in the Intestinal Tract. Front Immunol. 2020;11:575. 10.3389/fimmu.2020.00575. 32318067 PMC7147503

[5] Rinninella E, Raoul P, Cintoni M, Franceschi F, Miggiano GAD, Gasbarrini A, Mele MC. What is the healthy gut microbiota composition? A Changing ecosystem across age, environment, diet, and diseases. Microorganisms. 2019. 10.3390/microorganisms7010014 PMC6351938 30634578

[6] Guevarra RB, Hong SH, Cho JH, Kim B-R, Shin J, Lee JH, et al. The dynamics of the piglet gut microbiome during the weaning transition in association with health and nutrition. J Anim Sci Biotechnol. 2018;9:54. 10.1186/s40104-018-0269-6. 30069307 PMC6065057

[7] Tang X, Xiong K, Fang R, Li M. Weaning stress and intestinal health of piglets: A review. Front Immunol. 2022;13:1042778. 10.3389/fimmu.2022.1042778. 36505434 PMC9730250

[8] Ma N, Sun Y, Chen J, Qi Z, Liu C, Ma X. Micro-Coevolution of Genetics Rather Than Diet With Enterotype in Pigs. Front Nutr. 2022. 10.3389/fnut.2022.846974. 35392290 PMC8982514

[9] Tang W, Chen D, Yu B, He J, Huang Z, Zheng P, et al. Capsulized faecal microbiota transplantation ameliorates post-weaning diarrhoea by modulating the gut microbiota in piglets. Vet Res. 2020;51:55. 10.1186/s13567-020-00779-9. 32299514 PMC7164362

[10] Zha A, Tu R, Qi M, Wang J, Tan B, Liao P, et al. Mannan oligosaccharides selenium ameliorates intestinal mucosal barrier, and regulate intestinal microbiota to prevent Enterotoxigenic Escherichia coli -induced diarrhea in weaned piglets. Ecotoxicol Environ Saf. 2023;264:115448. 10.1016/j.ecoenv.2023.115448. 37696080

[11] Zhang LL, Wu ZC, Li JY, Li HK, Liu ZM, Wang J, Tan BE. Ningxiang pig-derived Enterococcus hirae HNAU0516 ameliorates postweaning diarrhoea by promoting intestinal health and modulating the gut microbiota in piglets. Animal. 2024;18:101220. 10.1016/j.animal.2024.101220. 39213909

[12] Zhang L, Wu Z, Kang M, Wang J, Tan B. Utilization of Ningxiang pig milk oligosaccharides by Akkermansia muciniphila in vitro fermentation: enhancing neonatal piglet survival. Front Microbiol. 2024;15:1430276. 10.3389/fmicb.2024.1430276. 38933035 PMC11199860

[13] Cani PD, Delzenne NM. The role of the gut microbiota in energy metabolism and metabolic disease. Curr Pharm Des. 2009;15:1546–58. 10.2174/138161209788168164. 19442172

[14] Yu LC-H, Wang J-T, Wei S-C, Ni Y-H. Host-microbial interactions and regulation of intestinal epithelial barrier function: From physiology to pathology. World J Gastrointest Pathophysiol. 2012;3:27–43. 10.4291/wjgp.v3.i1.27. 22368784 PMC3284523

[15] Ezeji JC, Sarikonda DK, Hopperton A, Erkkila HL, Cohen DE, Martinez SP, et al. Parabacteroides distasonis: intriguing aerotolerant gut anaerobe with emerging antimicrobial resistance and pathogenic and probiotic roles in human health. Gut Microbes. 2021;13:1922241. 10.1080/19490976.2021.1922241. 34196581 PMC8253142

[16] de Cruz P, Kang S, Wagner J, Buckley M, Sim WH, Prideaux L, et al. Association between specific mucosa-associated microbiota in Crohn’s disease at the time of resection and subsequent disease recurrence: a pilot study. J Gastroenterol Hepatol. 2015;30:268–78. 10.1111/jgh.12694. 25087692

[17] Sun H, Guo Y, Wang H, Yin A, Hu J, Yuan T, et al. Gut commensal Parabacteroides distasonis alleviates inflammatory arthritis. Gut. 2023;72:1664–77. 10.1136/gutjnl-2022-327756. 36604114

[18] Wang K, Liao M, Zhou N, Bao L, Ma K, Zheng Z, et al. Parabacteroides distasonis Alleviates Obesity and Metabolic Dysfunctions via Production of Succinate and Secondary Bile Acids. Cell Rep. 2019;26:222–e2355. 10.1016/j.celrep.2018.12.028. 30605678

[19] Sun Y, Nie Q, Zhang S, He H, Zuo S, Chen C, et al. Parabacteroides distasonis ameliorates insulin resistance via activation of intestinal GPR109a. Nat Commun. 2023. 10.1038/s41467-023-43622-3. 38007572 PMC10676405

[20] Feng X, Liu Y, Xu S, Ma J, Yuan H, Wang H, et al. Functional analysis of Parabacteroides distasonis F4: a novel probiotic strain linked to calf growth and rumen fermentation. J Anim Sci Biotechnol. 2025;16:50. 10.1186/s40104-025-01182-0. 40181465 PMC11969818

[21] Zhang K, Shen X, Han L, Wang M, Lian S, Wang K, Li C. Effects on the intestinal morphology, inflammatory response and microflora in piglets challenged with enterotoxigenic Escherichia coli K88. Res Vet Sci. 2023;157:50–61. 10.1016/j.rvsc.2023.02.011. 36871456

[22] Zhang L, Guo T, Zhan N, Sun T, Shan A. Effects of the antimicrobial peptide WK3 on diarrhea, growth performance and intestinal health of weaned piglets challenged with enterotoxigenic Escherichia coli K88. Food Nutr Res. 2021. 10.29219/fnr.v65.3448. 34262420 PMC8254467

[23] Wang Y, Zhang Z, Du M, Ji X, Liu X, Zhao C, et al. Berberine alleviates ETEC-induced intestinal inflammation and oxidative stress damage by optimizing intestinal microbial composition in a weaned piglet model. Front Immunol. 2024;15:1460127. 10.3389/fimmu.2024.1460127. 39351242 PMC11440249

[24] Smith F, Clark JE, Overman BL, Tozel CC, Huang JH, Rivier JEF, et al. Early weaning stress impairs development of mucosal barrier function in the porcine intestine. Am J Physiol Gastrointest Liver Physiol. 2010;298:G352–63. 10.1152/ajpgi.00081.2009. 19926814 PMC2838512

[25] Hu J, Chen L, Zheng W, Shi M, Liu L, Xie C, et al. Lactobacillus frumenti Facilitates Intestinal Epithelial Barrier Function Maintenance in Early-Weaned Piglets. Front Microbiol. 2018;9:897. 10.3389/fmicb.2018.00897. 29867808 PMC5958209

[26] Guerrant RL, DeBoer MD, Moore SR, Scharf RJ, Lima AAM. The impoverished gut–a triple burden of diarrhoea, stunting and chronic disease. Nat Rev Gastroenterol Hepatol. 2013;10:220–9. 10.1038/nrgastro.2012.239. 23229327 PMC3617052

[27] McKenzie R, Bourgeois AL, Frech SA, Flyer DC, Bloom A, Kazempour K, Glenn GM. Transcutaneous immunization with the heat-labile toxin (LT) of enterotoxigenic Escherichia coli (ETEC): protective efficacy in a double-blind, placebo-controlled challenge study. Vaccine. 2007;25:3684–91. 10.1016/j.vaccine.2007.01.043. 17313998

[28] Zhao L, Luo L, Jia W, Xiao J, Huang G, Tian G, et al. Serum diamine oxidase as a hemorrhagic shock biomarker in a rabbit model. PLoS ONE. 2014;9:e102285. 10.1371/journal.pone.0102285. 25144315 PMC4140717

[29] Martel J, Chang S-H, Ko Y-F, Hwang T-L, Young JD, Ojcius DM. Gut barrier disruption and chronic disease. Trends Endocrinol Metab. 2022;33:247–65. 10.1016/j.tem.2022.01.002. 35151560

[30] Koh GY, Kane AV, Wu X, Crott JW. Parabacteroides distasonis attenuates tumorigenesis, modulates inflammatory markers and promotes intestinal barrier integrity in azoxymethane-treated A/J mice. Carcinogenesis. 2020;41:909–17. 10.1093/carcin/bgaa018. 32115637

[31] Cuffaro B, Assohoun ALW, Boutillier D, Súkeníková L, Desramaut J, Boudebbouze S, et al. In Vitro Characterization of Gut Microbiota-Derived Commensal Strains: Selection of Parabacteroides distasonis Strains Alleviating TNBS-Induced Colitis in Mice. Cells. 2020. 10.3390/cells9092104. 32947881 PMC7565435

[32] Meyer F, Wendling D, Demougeot C, Prati C, Verhoeven F. Cytokines and intestinal epithelial permeability: A systematic review. Autoimmun Rev. 2023;22:103331. 10.1016/j.autrev.2023.103331. 37030338

[33] Liu G, Liu H, Tian W, Liu C, Yang H, Wang H, et al. Dietary nucleotides influences intestinal barrier function, immune responses and microbiota in 3-day-old weaned piglets. Int Immunopharmacol. 2023;117:109888. 10.1016/j.intimp.2023.109888. 36827918

[34] Koh GY, Kane A, Lee K, Xu Q, Wu X, Roper J, et al. Parabacteroides distasonis attenuates toll-like receptor 4 signaling and Akt activation and blocks colon tumor formation in high-fat diet-fed azoxymethane-treated mice. Int J Cancer. 2018;143:1797–805. 10.1002/ijc.31559. 29696632

[35] Liu D, Zhang S, Li S, Zhang Q, Cai Y, Li P, et al. Indoleacrylic acid produced by Parabacteroides distasonis alleviates type 2 diabetes via activation of AhR to repair intestinal barrier. BMC Biol. 2023;21:90. 10.1186/s12915-023-01578-2. 37072819 PMC10114473

[36] Kverka M, Zakostelska Z, Klimesova K, Sokol D, Hudcovic T, Hrncir T, et al. Oral administration of Parabacteroides distasonis antigens attenuates experimental murine colitis through modulation of immunity and microbiota composition. Clin Exp Immunol. 2011;163:250–9. 10.1111/j.1365-2249.2010.04286.x. 21087444 PMC3043316

[37] Brooks SA, Blackshear PJ. Tristetraprolin (TTP): interactions with mRNA and proteins, and current thoughts on mechanisms of action. Biochim Biophys Acta. 2013;1829:666–79. 10.1016/j.bbagrm.2013.02.003. 23428348 PMC3752887

[38] Cao S, Hou L, Sun L, Gao J, Gao K, Yang X, et al. Intestinal morphology and immune profiles are altered in piglets by early-weaning. Int Immunopharmacol. 2022;105:108520. 10.1016/j.intimp.2022.108520. 35063748

[39] Wu H, Xie S, Miao J, Li Y, Wang Z, Wang M, Yu Q. Lactobacillus reuteri maintains intestinal epithelial regeneration and repairs damaged intestinal mucosa. Gut Microbes. 2020;11:997–1014. 10.1080/19490976.2020.1734423. 32138622 PMC7524370

[40] Zhu X, Yang M, Lin Z, Mael SK, Li Y, Zhang L, et al. REGγ drives Lgr5 + stem cells to potentiate radiation induced intestinal regeneration. Sci China Life Sci. 2022;65:1608–23. 10.1007/s11427-021-2018-7. 34826093

[41] Arijs I, de Hertogh G, Lemaire K, Quintens R, van Lommel L, van Steen K, et al. Mucosal Gene Expression of Antimicrobial Peptides in Inflammatory Bowel Disease Before and After First Infliximab Treatment. PLoS ONE. 2009;4:e7984. 10.1371/journal.pone.0007984. 19956723 PMC2776509

[42] Dong Y, Fan H, Zhang Z, Jiang F, Li M, Zhou H, et al. Berberine ameliorates DSS-induced intestinal mucosal barrier dysfunction through microbiota-dependence and Wnt/β-catenin pathway. Int J Biol Sci. 2022;18:1381–97. 10.7150/ijbs.65476. 35280677 PMC8898376

[43] Muntjewerff EM, Tang K, Lutter L, Christoffersson G, Nicolasen MJT, Gao H, et al. Chromogranin A regulates gut permeability via the antagonistic actions of its proteolytic peptides. Acta Physiol (Oxf). 2021;232:e13655. 10.1111/apha.13655. 33783968 PMC8341099

[44] Hildebrand F, Nguyen TLA, Brinkman B, Yunta RG, Cauwe B, Vandenabeele P, et al. Inflammation-associated enterotypes, host genotype, cage and inter-individual effects drive gut microbiota variation in common laboratory mice. Genome Biol. 2013;14:R4. 10.1186/gb-2013-14-1-r4. 23347395 PMC4053703

[45] Messori S, Trevisi P, Simongiovanni A, Priori D, Bosi P. Effect of susceptibility to enterotoxigenic Escherichia coli F4 and of dietary tryptophan on gut microbiota diversity observed in healthy young pigs. Vet Microbiol. 2013;162:173–9. 10.1016/j.vetmic.2012.09.001. 23021862

[46] Wang W, Wang Y, Hao X, Duan Y, Meng Z, An X, Qi J. Dietary fermented soybean meal replacement alleviates diarrhea in weaned piglets challenged with enterotoxigenic Escherichia coli K88 by modulating inflammatory cytokine levels and cecal microbiota composition. BMC Vet Res. 2020;16:245. 10.1186/s12917-020-02466-5. 32664940 PMC7362456

[47] Li J, Feng S, Wang Z, He J, Zhang Z, Zou H, et al. Limosilactobacillus mucosae-derived extracellular vesicles modulates macrophage phenotype and orchestrates gut homeostasis in a diarrheal piglet model. NPJ Biofilms Microbiomes. 2023;9:33. 10.1038/s41522-023-00403-6. 37280255 PMC10244441

[48] Eshleman EM, Alenghat T. Epithelial sensing of microbiota-derived signals. Genes Immun. 2021;22:237–46. 10.1038/s41435-021-00124-w. 33824498 PMC8492766

[49] Mukhopadhya I, Louis P. Gut microbiota-derived short-chain fatty acids and their role in human health and disease. Nat Rev Microbiol. 2025;23:635–51. 10.1038/s41579-025-01183-w. 40360779

[50] Shen Y, Qu W, Song M, Zhang T, Liu C, Shi X, et al. Single-microbe RNA sequencing uncovers unexplored specialized metabolic functions of keystone species in the human gut. iMeta. 2025;4:e70035. 10.1002/imt2.70035. 40469507 PMC12130570

