# Supplementary material for: Parabacteroides distasonis ameliorates ETEC-induced diarrhea in weaned piglets by regulating intestinal barrier function and intestinal inflammation
Source: Porcine Health Manag. 2026 Jun 18;12:49. doi: 10.1186/s40813-026-00525-1 (PMC13527943; doi:10.1186/s40813-026-00525-1)
Supplement: Supplementary file 1 [file 40813_2026_525_MOESM1_ESM.docx]

Supplementary Table S1 Basic diet composition and nutritional component (%, as-fed basis)

| Ingredients | Contents (%) | Nutrients |  |
| --- | --- | --- | --- |
| Corn | 62.00 | Digest energy, MJ/kg | 14.30 |
| Soybean meal | 20.00 | Crude protein, % | 18.25 |
| Fish meal | 5.00 | Ether extract, % | 6.01 |
| Whey powder | 5.00 |  |  |
| Soybean oil | 3.00 |  |  |
| Dicalcium Phosphate | 1.00 |  |  |
| Lysine | 0.80 |  |  |
| White sugar | 0.70 |  |  |
| Limestone | 0.60 |  |  |
| Acidifier ^2^ | 0.50 |  |  |
| Threonine | 0.30 |  |  |
| Salt | 0.30 |  |  |
| Choline | 0.16 |  |  |
| Methionine | 0.10 |  |  |
| Zinc oxide | 0.10 |  |  |
| Premix ^1^ | 0.10 |  |  |
| Antioxidants premix ^2^ | 0.10 |  |  |
| Copper sulfate pentahydrate | 0.07 |  |  |
| Zinc sulfate pentahydrate | 0.05 |  |  |
| Fungicide ^2^ | 0.05 |  |  |
| Fragrance | 0.05 |  |  |
| Sweetener | 0.02 |  |  |

^1 The premix provided the following per kilogram of diets: vitamin A 24,375 IU, vitamin B1 7.5 mg, vitamin B2 18.75 μg, vitamin B6 9 mg, vitamin B12 75 mg, vitamin D3 7,500 IU, vitamin E 60 IU, vitamin K3 3 7.5 mg, biotin 0.45 mg, folic acid 1.0 mg, D-pantothenic acid 37.5 mg, nicotinic acid 75 mg, Cu (as copper sulfate) 6 mg, Fe (as ferrous sulfate) 100 mg, Mn (as manganese sulfate) 4 mg, Zn (as zinc sulfate) 100 mg, I (as potassium iodide) 0.14 mg, Se (as sodium selenite) 0.30 mg.^

^2 The product for acidifier is named Zhonghua dynamic acid type I (active compound: lactic acid, citric acid and hawthorn). The product for antioxidant premix is named Antioxidant-33 (active compound: ethoxyquinoline). The product for fungicide is named plant essential oils (active compound: thymol, carvonol, and cinnamaldehyde).^

**Supplementary Table S2 Primers used for gene expression analysis by real-time PCR**

| Number | Gene | Primer sequence(5’-3’) |
| --- | --- | --- |
| 1 | *Claudin-1* | F: GCATCATTTCCTCCCTGTT  R: TCTTGGCTTTGGGTGGTT |
| 2 | *Claudin-3* | F：CATTATCACAGCGCGGATCA  R：CGTACACTTTGCACTGCATCTG |
| 3 | *Occludin* | F: CAGTGGTAACTTGGAGGCGTCTTC  R: CGTCGTGTAGTCTGTCTCGTAATGG |
| 4 | *ZO-1* | F: TTGATAGTGGCGTTGACA  R: CCTCATCTTCATCATCTTCTAC |
| 5 | *INF-γ* | F:TTCAGCTTTGCGTGACTTTG  R:GGTCCACCATTAGGTACATCTG |
| 6 | *IL-1β* | F: CCTGGACCTTGGTTCTCT  R: GGATTCTTCATCGGCTTCT |
| 7 | *IL-10* | F: TCGGCCCAGTGAAGAGTTTC  R: GGAGTTCACGTGCTCCTTGA |
| 8 | *TNF-α* | F: ATCGGCCCCCAGAAGGAAGAG  R: GATGGCAGAGAGGAGGTTGAC |
| 9 | *IL-6* | F: CCCAACTTCCAATGCTCTCCT  R: GGATGGTCTTGGTCCTTAGCC |
| 10 | *IL-8* | F: AGTTTTCCTGCTTTCTGCAGCT  R: TGGCATCGAAGTTCTGCACT |
| 11 | *TGF-β* | F:CGGACTACTACGCCAAGGAG  R:CGCCAGGAATCATTGCCTGTATT |
| 12 | *PCNA* | F:TACGCTAAGGGCAGAAGATAATG  R: CTGAGATCTCGGCATATACGTG |
| 13 | *Lgr5* | F:GCCTTTGTAGGCAACCCTTC  R: AGGCACCATTCAAAGTCAGTG |
| 14 | *CHGA* | F:CAGACCCCCGACTTTAAACCA  R:TTGTGCCCAGTGGCTCTAAT |
| 15 | *LYZ* | F: CCCGGCTTCTCAGACAACAT  R: CCTATAGCCGTCCATGCCAG |
| 16 | *β-actin* | F: CTGCGGCATCCACGAAACT  R: AGGGCCGTGATCTCCTTCTG |

**Supplementary Figures**

**Figure S1**. (A) The concentration of feces sIgA. (B) The concentration of feces DEFβ2. (C) Correlation analysis between fecal immune indexes (sIgA, DEFβ2) and fecal microbiota (day 14). Data are presented as the Mean ± SME. “*” indicated 0.1 < *P* ≤ 0.05, “**” indicated 0.05 < *P* ≤ 0.01.

**Figure S2**.(A) The relative abundance of ileal contents ETEC was determined by q-PCR. (B-D) Statistical analysis of intestinal villi and crypts, values are expressed in micrometers (μm). (E-H) Relative mRNA expressions of *Claudin-1*, *Claudin-3*, *Occludin*, and *ZO-1*in ileum of piglets (n = 6). Data are presented as the Mean ± SME. “*” 0.1 < *P* ≤ 0.05, “**” 0.05 < *P* ≤ 0.01, “***”*P* < 0.001.
